# Predicting **β**-Turns in Protein Using Kernel Logistic Regression

**DOI:** 10.1155/2013/870372

**Published:** 2013-02-19

**Authors:** Murtada Khalafallah Elbashir, Yu Sheng, Jianxin Wang, FangXiang Wu, Min Li

**Affiliations:** ^1^School of Information Science and Engineering, Central South University, Changsha 410083, China; ^2^Department of Mechanical Engineering, University of Saskatchewan, Saskatoon, SK, Canada S7N 5A9

## Abstract

A **β**-turn is a secondary protein structure type that plays a significant role in protein configuration and function. On average 25% of amino acids in protein structures are located in **β**-turns. It is very important to develope an accurate and efficient method for **β**-turns prediction. Most of the current successful **β**-turns prediction methods use support vector machines (SVMs) or neural networks (NNs). The kernel logistic regression (KLR) is a powerful classification technique that has been applied successfully in many classification problems. However, it is often not found in **β**-turns classification, mainly because it is computationally expensive. In this paper, we used KLR to obtain sparse **β**-turns prediction in short evolution time. Secondary structure information and position-specific scoring matrices (PSSMs) are utilized as input features. We achieved *Q*_total_ of 80.7% and MCC of 50% on BT426 dataset. These results show that KLR method with the right algorithm can yield performance equivalent to or even better than NNs and SVMs in **β**-turns prediction. In addition, KLR yields probabilistic outcome and has a well-defined extension to multiclass case.

## 1. Introduction

 The number of known protein sequence is increasing rapidly as a result of genome and other sequencing projects. Consequently, this increase widens sequence-structure gap rapidly [1, 2]. Thus computational tools for predicting protein structure and function are highly needed to narrow the widening gap [3]. There are four distinct levels of protein structures. These levels are primary structure which refers to amino acid linear sequence of the polypeptide, secondary structure, which is defined by the patterns of hydrogen bonds between backbone amide and carboxyl groups, tertiary structure, which is the three-dimensional structure of a single protein molecule, and quaternary structure, which is a larger assembly of several protein molecules or polypeptide chains.

The basic elements of the secondary structure of proteins are *α*-helices, *β*-sheets, coils, and turns. A turn is a structural motif where the *α*-atoms of two residues are separated by few (usually from 1 to 5) peptide bonds, and the distance between them is less than 7*A*°, while the corresponding residues do not form a regular secondary structure element such as an *α*-helix or *β*-sheet. Different turns are classified according to the separation between the two end residues. The end residues are separated by four peptide bonds in *α*-turns, three peptide bonds in *β*-turns, two peptide bonds in *γ*-turns, one bond in *δ*-turns, and five bonds in *π*-turns. *β*-turns are the most common found type of turns that constitute approximately 25% of the residues in protein. They play a significant role in protein configuration and function, and its formation is a vital stage during the protein folding. They were found to be more helpful in the context of molecular recognition and in modeling interactions between peptide substrates receptors, because they tend to be more solvent exposed than buried [4]. In the recent years it has been found that *β*-turns are important in the design of various peptidomimetics for many diseases [5]. Therefore, development of effective and efficient prediction methods for *β*-turns identification in protein is useful in fold recognition and drug design [6].

*β*-turns are further classified into different types according to the dihedral angles (*φ*, *ψ*) of the central two residues. The classification scheme proposed by Hutchinson and Thornton [7] recognizes nine distinct types of *β*-turn: I, I′, II, II′, VIa1, VIa2, VIb, VIII, and IV. In this classification, the most frequently occurring type is type IV, which constitutes approximately (35%) of the *β*-turns. Types VIa1,VIa2, and VIb are rare types.

Most of the successful *β*-turns prediction methods are based on either support vector machines (SVMs) or neural networks (NNs). Zheng and Kurgan [6] applied SVM-based ensemble to predict *β*-turns. They used position-specific scoring matrices (PSSMs) and secondary structure information as features in their prediction model. Kountouris and Hirst [8] developed a method based on SVM; their method uses PSSMs, predicted secondary structures, and predicted dihedral angles as input features to the SVM. Shepherd et al. [9] used a neural network to predict both the location and types of *β*-turn in protein; they incorporated secondary structure information on the features used as input to the NN. Kaur and Raghava [10] used two feedforward backpropagation networks with a single hidden layer, where the first sequence-structure network is trained with the PSSMs. The initial prediction from the first network and the predicted secondary structure using PSIPRED [11, 12] are used as input to the second structure-structure network to refine the prediction obtained from the first network. Petersen et al. [13] presented a neural network method called NetTurnP, for predicting *β*-turns and *β*-turn types. Their method consists of two artificial neural network layers; they used PSSMs, secondary structure, and surface accessibility as input to their model.

There is another method that can perform well as SVMs and NNs, which is the Kernel Logistic Regression (KLR). KLR is a kernel version of logistic regression (LR). It is often not found in predicting protein secondary structures and *β*-turns due to its computational demand. However, unlike SVMs and NNs, KLR yields a posteriori probabilities based on a maximum likelihood argument that is, besides predicting class labels, KLR provides interpretation about this labeling. When it comes to *β*-turn types prediction, KLR has an additional advantage that its extension to multiclass classification is well described. Karsmakers [14, 15] proposed a fast and accurate approximate implementation of KLR for Automatic Speech Recognition (ASR). He described a different practical technique suited for large datasets, based on fixed-size least squares support vector machines (FS-LSSVMs), of which he named fixed-size kernel logistic regression (FS-KLR). Karsmakers used trust region Newton's method for large-scale LR [16] as a basis to solve the approximate problem and Nystrom method to approximate the features' matrix. In this paper, we show that FS-KLR can be used in predicting *β*-turns in an efficient and effective way, and it yields results that are comparable to the state-of-the-art methods.

## 2. Methods

### 2.1. Data Sets

 The uniform dataset of 426 nonhomologous proteins (BT426) [17], the dataset of 547 protein sequence (BT547), and the dataset of 823 protein sequence (BT823) are used to evaluate the performance of our KLR method. Several researchers used BT426 as a golden set of sequences upon which performance values are reported and compared. This dataset consists of protein chains whose structure has been determined by X-ray crystallography at a resolution of <2.0*A*° or better. Each chain contains at least one *β*-turns region. In total 23,580 amino acids, corresponding to 24.9% of all amino acids, have been assigned to be located in *β*-turns. None of the sequences in the dataset shares more than 25% sequence identity. BT426 has been used by various recent *β*-turns prediction methods; therefore, we can use it to make direct comparisons with these methods. The other two datasets: BT547 and BT823 are constructed for training and testing COUDES [18].

### 2.2. Features Vector

The features that are used in this study include PSSMs and secondary structure information.

#### 2.2.1. PSSMs

Several studies show that PSSMs contributed significantly to the accuracy of *β*-turns prediction [6, 13]. The PSSMs are in the form of 20∗*M*, where*M*represents the sequence length. The PSSMs were generated using the iterative PSI-BLAST program [19] against National Center for Biotechnology Information (NCBI) nonredundant (nr) sequence database using the default parameters. The PSSMs values are scaled to values between 0 and 1. A window size of seven residues is used for the PSSMs. This is in accordance with Shepherd et al. [9] who found that the optimal prediction for *β*-turns is achieved using window size of seven or nine. The total number of the features that are based on PSSMs is (20∗7 = 140).

#### 2.2.2. Secondary Structure Information

For the secondary structure information features, four secondary structure prediction methods are utilized for all protein chains. These four prediction methods are PSIPRED [12, 20], JNET [21], TRANSEC [22], and PROTEUS [22]. The secondary structures were predicted as three structure states: helix (*H*), strand (*E*), and coils (*C*). These three structure states are encoded as 1 0 0 for helix, 0 1 0 for strand, and 0 0 1 for coils. The secondary structure information features are organized as follows: (1) a binary value denoting the prediction of a given secondary structure method from the aforementioned used prediction methods for the central residue; that is, if PSIPRED predicted the central amino acid to be helix, JNET predicted it to be coil, TRANSEC predicted it to be helix, and PROTEUS predicted it to be helix, then this binary value will be 1 0 0 0 0 1 1 0 0 1 0 0, so the total number of features using this organization is 12; (2) the confidence value obtained from the central residue using the four prediction methods. The confidence score is divided by 10 to normalize it to a unit interval, and the total number of features using this organization is 4. (3) A binary value denoting a specific configuration of the secondary structure is predicted using the four prediction methods for the central and the two adjacent residues. Here we have four patterns 1, 2, 3, and 4. If the predicted secondary structure using specific method is coils 0 0 1, the secondary structure for the pattern 1 will be CCC and for pattern 2, 3, and 4 will be CCX, XCC, and XCX, respectively, where *X* = *E*, *H*. The total number of features based on this organization is (4 patterns ∗ 3 secondary structures ∗ 4 prediction methods = 48 features). (4) The ratio between the number of residues in a given secondary structures and the window size for the four prediction methods, the number of features based on this organization will be (3 secondary structure ∗ 4 prediction methods = 12). The total number of features based on secondary structure information is 76. The motivation to use this organization comes from [6].

**Figure 1:**
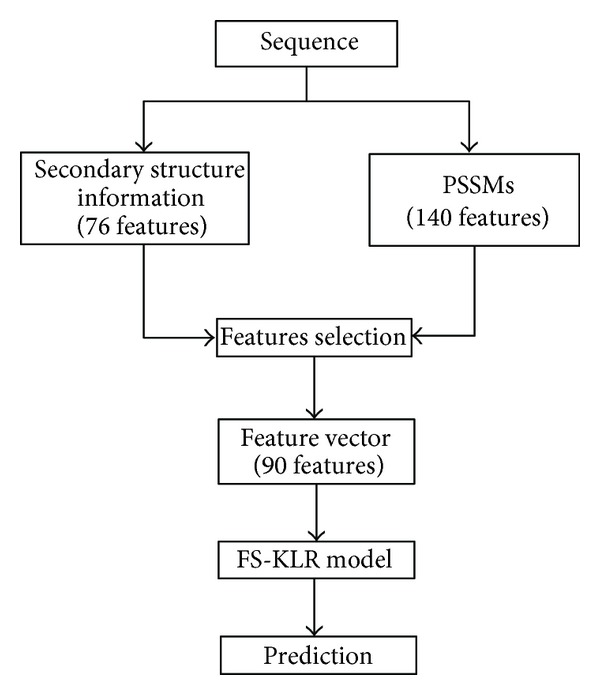
The architecture of the KLR method.

The predicted secondary structure information is added to the PSSMs features. The total number of the features that are based on PSSMs and secondary structure information is 216. Similarly as in [6], feature's selection methods based on information gain and CHI squared are employed to reduce the number of features to 90 features.  Figure 1 shows the overall architecture of our KLR method.

### 2.3. Prediction Method

The fixed-size kernel logistic regression (FS-KLR) was applied to predict *β*-turns. KLR is the kernel version of LR, which is a well-known statistical model for classification. Unlike LR, KLR enables the classification of linearly nonseparable problems by transferring the input features to a higher-dimensional space, via the kernel trick. The kernel is a transformation function that must satisfy Mercer's necessary and sufficient conditions, which state that a kernel function must be expressed as an inner product and must be positive semidefinite. Similar to LR, KLR can be fitted using the maximum likelihood estimate (MLE).

Iteratively reweighted least square (IRLS) algorithm is one of the most popular techniques used to find the MLE of the LR models. IRLS is a nonlinear optimization algorithm that uses a series of weighted least squares (WLS) sub-problems to search for the MLE. It is a special case of Fisher's scoring method, a quasi-Newton algorithm that replaces the objective function's Hessian with the Fisher information. For LR, IRLS is a special form of Newton's method in which each iteration finds the WLS estimates for a given set of weights, which are used to construct a new set of weights. KLR also can be fitted effectively using IRLS [15].

Unlike SVMs, KLR does not use risk minimization principle, but it is based on conditional maximum likelihood inference, which results in estimates of a posteriori class probabilities via logit stochastic models:

(1) $$\begin{array}{c} P\left(Y=-1\;|\;X=x;f\right)=\frac{\exp\left(f\left(x\right)\right)}{1+\exp\left(f\left(x\right)\right)},\\ P\left(Y=1\;|\;X=x;f\right)=\frac{1}{1+\exp\left(f\left(x\right)\right)}, \end{array}$$

where *f*(*x*) = *w* ^*T*^ *φ*(*x*) + *b*, *w* is the vector of the KLR parameters, and *b* is the intercept. The penalized negative log likelihood (PNLL) is normally used to infer the parameters of the KLR model. In the primal weight space, the objective function for the PNLL is as follows:

(2) $$\begin{array}{c} \underset{w,b}{\min}\frac{1}{2}w^{T}w+\frac{\lambda }{2}\sum_{i=1}^{N}\log\left(1+\exp\left(-y_{i}f\left(x_{i}\right)\right)\right), \end{array}$$

where *λ* is the regularization parameter. The solution *w* can be expressed in terms of *α* and computed using IRLS iteration as

(3) $$\begin{array}{c} w=\sum_{i=1}^{N}\alpha_{i}\varphi \left(x_{i}\right). \end{array}$$

In the dual representation, the function values *f*(*x*) in the KLR logit models can be computed as follows:

(4) $$\begin{array}{c} f\left(x\right)=\sum_{i=1}^{N}\alpha_{i}K\left(x,x_{i}\right)+b, \end{array}$$

where  *K*(*x*, *x*_*i*_) = *φ*(*x*)^*T*^ *φ*(*x*_*i*_).

The IRLS method is suitable for small size problems, but for large-scale problems this method becomes computationally expensive. Based on fixed-size least squares support vector machines (FS-LSSVMs) Karsmakers [14, 15] implemented a fixed-size variant of the standard KLR formulation (FS-KLR) which does easily scale to very large datasets. In his method, he adopted Nystrom approximation method. 

In Nystrom approximation, the kernel matrix will be decomposed into eigenvalues/eigenvectors matrices in the form:

(5) $$\begin{array}{c} K_{n\times n}=U_{n}\Lambda_{n}{U_{n}}^{T}, \end{array}$$

where Λ_*n*_ = diag(*λ*_*i*_) and *λ*_1_ ≥ *λ*_2_ ≥ ⋯≥*λ*_*n*_ ≥ 0 are the eigenvalues of the matrix *K*, *U*_*n*_ is the matrix of the eigenvectors that correspond to the eigenvalues, and *n* is the number of the data points. We can select the first *p* eigenvectors and eigenvalues from the matrices *U* and Λ, respectively, where *p* ≪ *n*, to approximate the kernel matrix. This approximation is motivated by its widely usage, for example, principal component analysis. Using this approximation reduces the computational cost drastically. However, computing the eigendecomposition is also computationally expensive. To reduce the computational cost of computing the eigendecomposition we selected a small sample of size *m* from the features' matrix to construct the following eigen-problem:

(6) $$\begin{array}{c} K_{m\times m}=U_{m}\Lambda_{m}{U_{m}}^{T}. \end{array}$$

We can extend the eigenvalues/eigenvectors of the *K*_*m*×*m*_ to all the points using the following Nystrom approximation:

(7) $$\begin{array}{c} {\tilde{\lambda }}_{i}^{\left(n\right)}=\frac{n}{m}\lambda_{i}^{\left(m\right)},\\ {\tilde{u}}_{i}^{\left(n\right)}=\sqrt{\frac{m}{n}}\frac{1}{\lambda_{i}^{\left(m\right)}}K_{n,m}u_{i}^{\left(m\right)}, \end{array}$$

where *λ*_*i*_^(*m*)^ and *u*_*i*_^(*m*)^ are the *i*th eigenvalue/eigenvector of the *m* × *m* eigenproblem and *K*_*n*,*m*_ is the appropriate *n* × *m* submatrix of *K*.

The selected sample of size *m* from the features' matrix can be called prototype vectors (PVs). These PVs can be selected using *k*-center clustering. The use of *k*-center clustering is justified in [23], which observed that the Nystrom low-rank approximation depends crucially on the quantization error induced by encoding the sample set with landmark points. This suggests that one can simply use the clusters obtained with a *k*-center (such as *k*-means) algorithm, which finds a local minimum of the quantization error. The PVs selection methods using *k*-center clustering suffer from the fact that they will select outliers as prototypes. In cases where the number of PVs is relatively small, the fraction of prototypes chosen to represent the nonoutlier and outlier data is unbalanced, and, therefore, the classification performance will not be optimal. When the number of PVs is increased, the performance will also increase to that of KLR. Hence taking into account outliers removal can result in a sparser model. The sparse kernel logistic regression problem is solved in the primal space using Newton's trust region algorithm, which is given in [16]. This algorithm yielded the best performance compared to the state-of-the-art alternatives. Convergence speed and cost per iteration will be balanced in that low-cost approximate because Newton's steps will be taken in the beginning of the algorithm and full Newton directions at the end for fast convergence. In this paper, the following radial basis function (RBF) is used as a kernel function:

(8) $$\begin{array}{c} K\left(x_{i},x_{i}^{\prime }\right)=e^{-\gamma {\left|x_{i}-x_{i}^{\prime }\right|}^{2}}, \end{array}$$

where *γ* is the kernel parameter.

### 2.4. Model Selection

Model selection is the process of determining the optimal regularization parameter *λ* and the kernel parameter *γ*. It is a very important step in fitting kernel models to maximize generalization performance. The cross-validation-based method is used to determine the optimal parameters for *β*-turns prediction.

### 2.5. Training and Testing

 In order to evaluate a prediction method it is necessary to have different datasets for training and testing. The jackknife test is the most objective and rigorous cross-validation method compared with independent dataset test and subdataset test [24]. In a full jackknife test of *N* proteins, one protein is removed from the set; the training is done on the remaining *N* − 1 proteins, and the test is done on the removed protein. This process is repeated *N* times by removing one protein in turn. Since this training technique is very time consuming most of the recent *β*-turns prediction methods use sevenfold cross-validation to assess their performances. We also used sevenfold cross-validation to assess the accuracy of FS-KLR. In sevenfold cross-validation, the datasets will be divided into seven subsets, each containing equal number of proteins. Each set is an unbalanced set that retains the naturally occurring proportion of *β*-turns. Six of the seven subsets were merged together to form a training set that was used to train the FS-KLR methods, and the seventh was used for validation. This process was repeated seven times in order to have a different set for validation each time. The final prediction results are taken as the average of the results from the seven testing sets. 

### 2.6. Performance Measures

 The quality of prediction is evaluated using five measures: MCC, *Q*_total_, *Q*_predicted_, *Q*_observed_, and Specificity. These measures are consistent with the test procedures and measures applied to evaluate competing methods. Let (true positives) TP be the number of correctly classified *β*-turns residues, (true negatives) TN be the number of correctly classified non-*β*-turns residues, (false positives) FP be the number of non-*β*-turns incorrectly classified as *β*-turns residues, and (false negatives) FN be the number of *β*-turns incorrectly classified as non-*β*-turns residues. The Matthews correlation coefficient (MCC) can be calculated as [25]

(9) $$\begin{array}{c} \text{MCC}=\frac{\left(\text{TP}*\text{TN}-\text{FP}*\text{FN}\right)}{\sqrt{\left(\text{TP}+\text{FN}\right)*\left(\text{TN}+\text{FP}\right)*\left(\text{TP}+\text{FP}\right)*\left(\text{TN}+\text{FN}\right)}}. \end{array}$$

The result of MCC is in the range of −1 and 1, where a value of 1 indicates a perfect positive correlation, a value of −1 indicates a perfect negative correlation, and a value of 0 indicates no correlation.

*Q*_total_ (prediction accuracy), which is defined as the percentage of correctly classified residues, is calculated as follows:

(10) $$\begin{array}{c} Q_{\text{total}}=\frac{\text{TP}+\text{TN}}{\text{TP}+\text{TN}+\text{FP}+\text{FN}}\times 100. \end{array}$$

Probability of correct prediction or *Q*_predicted_ is the percentage of correctly predicted *β*-turns among the predicted *β*-turns. It is also called predicted positive value (PPV), and it is given as follows:

(11) $$\begin{array}{c} Q_{\text{predicted}}=\frac{\text{TP}}{\text{TP}+\text{FP}}\times 100. \end{array}$$

Sensitivity or coverage (also known as  *Q*_observed_) is the percentage of correctly predicted *β*-turns among the observed *β*-turns, or it is the fraction of the total positive samples that are correctly predicted, and it is given as follows:

(12) $$\begin{array}{c} Q_{\text{observed}}=\frac{\text{TP}}{\text{TP}+\text{FN}}\times 100. \end{array}$$

Specificity is the fraction of total negative samples that are correctly predicted

(13) $$\begin{array}{c} \text{Specificity}=\frac{\text{TN}}{\text{TN}+\text{FP}}\times 100. \end{array}$$

## 3. Results

**Figure 2:**
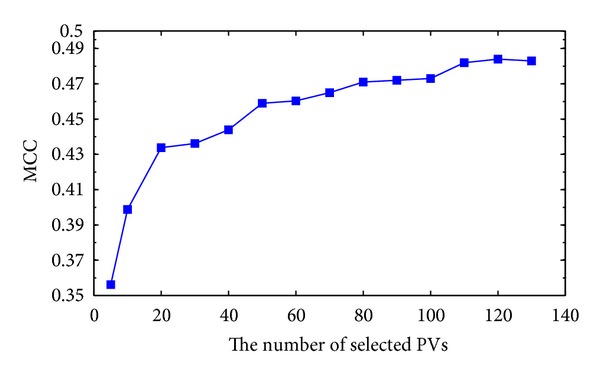
The MCC in function of the number of the selected PVs.

**Figure 3:**
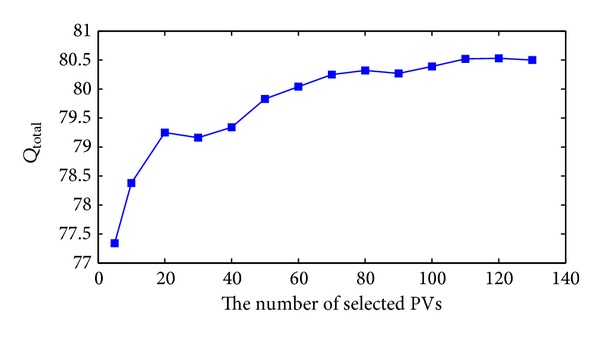
The *Q*_total_ in function of the number of the selected PVs.

 The selected number of Pvs (*m*) where *m* ≪ *n* from the feature matrix affects the accuracy and the MCC of the prediction. A relatively small or big *m* will yield low performance. To select the optimal number of vectors a cross-validation is used starting with relatively small *m* and adding more vectors to *m* until a point where adding more vectors does not improve the classification performance reached.  Table 1 shows the prediction accuracy and MCC using different values of *m*. In Figure 2, we see that the highest MCC is achieved for *m* equal 120, while Figure 3 shows that the highest accuracy is achieved using *m* equal to 115 or 120. The dataset used for the two figures is BT426.

After a short analysis of various values of threshold, we set its value to 0.45 to obtain the results in Table 1. The *Q*_total_ has improved slightly when the threshold value is set to 0.50, while the MCC dropped to less than 0.46. Similarly, the MCC has increased when the threshold value is set to 0.40, but at the cost of *Q*_total_, which has dropped to less than 79%. The number of selected vectors *m* in this research is set to 120 for BT426 dataset. Using this value for *m* we obtained a *Q*_total_ of 80.54%, MCC of 0.48, *Q*_predicted_ of 59%, *Q*_observed_ of 62%, and Specificity of 86%. The MCC is a robust and reliable performance measure that accounts for both overpredictions and underpredictions. A high MCC value indicates a high prediction performance. 

To increase the performance of our KLR model further we used state changing rules. In these rules we put in our consideration that *β*-turns occur in a group of at least four adjacent residues. After analyzing the results obtained by the KLR prediction, the state changing rules, which will make the prediction to be more *β*-turn like, are derived as follows.

1. Change isolated nonturn predictions to turn (i.e., tnt→ ttt).
2. Change isolated turn prediction to non-turn prediction (i.e., ntn→ nnn).
3. Change the residues that are neighboring two isolated turn predictions to turn (i.e., nttn→ tttt).
4. If there is isolated triplet of turns predictions, then change the adjacent nonturn prediction with the highest KLR probability output to turn (i.e., ntttn→ ttttn or ntttt).

The above rules should be executed in orders. After applying these rules, we obtained a better performance, where the MCC has increased from 0.48 to 0.50. 

Table 2 shows the comparison between our KLR method and other best existing *β*-turns prediction methods. Our KLR method achieves prediction accuracy *Q*_total_ = 80.7%, *Q*_predicted_ = 58.98%, *Q*_observed_ = 65.25%, sensitivity = 85.34%, and MCC = 0.50. We note that the *Q*_total_ of our method is 0.2% lower than the *Q*_total_ of BTNpred and E-SSpred, but because *β*-turns account for approximately 25% of the globular protein residues, *Q*_total_ is a poor measure by itself, as it is possible to achieve *Q*_total_ of 75% by predicting all residues to be non-*β*-turns. Instead, our method shows high MCC 0.50 compared to BTNpred 0.47 and E-SSpred 0.44. The NetturnP and our method have the highest MCC 0.50 among the other *β*-turns prediction methods. Other than BTNpred and E-SSpred our KLR shows the highest *Q*_total_. When combining *Q*_total_ and MCC our method has the highest performance among the other prediction methods. Considering the baseline accuracy which equals 75%, our method provides 5.7/25 = 0.23% error rate reduction, while BTNpred and E-SSpred provide 5.9/25 = 0.24% error rate reduction, and the second best method (SVM) provides 4.8/25 = 0.19% error rate reduction. The *Q*_observed_ of our method is higher by 9.65% than the *Q*_observed_ of BTNpred, by 3.25% than the *Q*_observed_ of the BTSVM, and by 16.05% than the *Q*_observed_ of E-SSpred. Higher *Q*_observed_ values mean that a large percentage of the observed *β*-urns are correctly predicted. At the same time, the *Q*_predicted_ of our method shows that 58% of the actual *β*-turns are correctly predicted. We note that the *Q*_predicted_ of our method is 3.72% lower than the *Q*_predicted_ of BTNpred and 4.62% lower than the *Q*_predicted_ of the E-SSpred. The increase in the *Q*_observed_ values is a tradeoff for the decrease in the *Q*_predicted_ values. In spite of this tradeoff, our method shows high overall accuracy.

Besides BT426 dataset that is used for training and testing our method, we used two additional datasets, that is, BT547 and BT823 datasets, to validate the performance of our method. Results obtained based on sevenfold cross-validation on these datasets are given in Table 3. The results show that for the BT547 dataset our method obtains *Q*_total_ = 80.46%, *Q*_predicted_ = 59.04%, *Q*_observed_ = 65.36%, and MCC = 0.50. The MCC of our method is the best among other competing methods that are evaluated on BT547 dataset. For the BT823 dataset our method obtains *Q*_total_ = 80.66%, *Q*_predicted_ = 58.42%, *Q*_observed_ = 64.64%, and MCC = 0.49. Also our method has the highest MCC on BT547 and BT823 datasets. The results also show that our method shows stable performances on all the three datasets used. 

**Figure 4:**
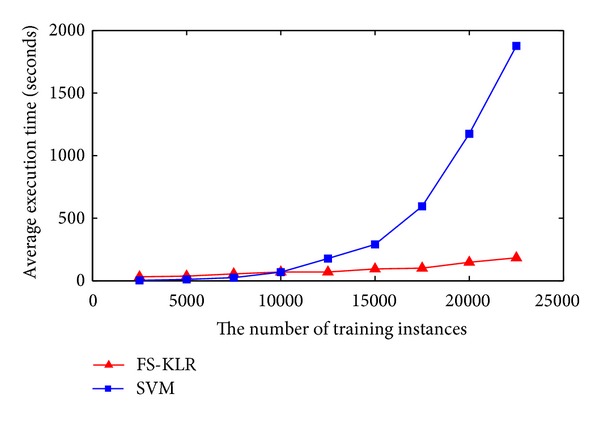
Average execution time of the KLR model in function of the number of the training instances.

All the computations for KLR were carried out using Matlab version 2010b on a computer with 3 GB RAM and 1.86 GHz Genuine Intel dual core processor. We compared the average elapsed time of our method with the BTNpred and E-SSpred. The results of the comparison are shown in Table 4. In this comparison, we used onefold out of the sevenfolds in all the datasets as a test set and the remaining folds as training set. Since both BTNpred and E-SSpred used SVM, we used LIBSVM [30] on their features. Note that both E-SSpred and BTNpred used PSSMs and secondary structure information as features. In addition to PSSMs and secondary structure information, E-SSpred added amino acid (AA) composition generated with classical local coding scheme. We also compared the average execution time of the KLR method with LIBSVM on our feature vector using BT426 dataset.  Figure 4 shows the average execution time of the KLR method and LIBSVM in function of the number of the training instances.

Compared to E-SSpred and BTNpred as shown in Table 4, our method is faster by more than a factor of 14. Although the training data in BT823 is more than the training data in BT547, its computation time using KLR is less than the computation time of BT547, that is because the number of selected vectors *m* for BT823 is 90, which is by far less than the number of selected vectors for BT547, which is 140. This indicates that, for a very large dataset, a very small number of selected vectors *m* can be sufficient to approximate its Kernel matrix, which reflects the capability of the FS-KLR model to handle large-scale datasets. 

**Figure 5:**
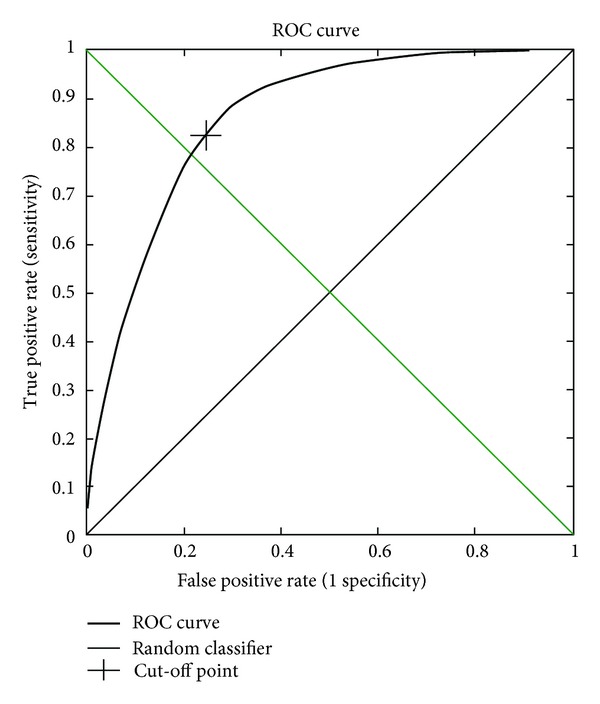
ROC curve for the evaluation of the KLR model on the BT426 dataset.

The ROC curve, which is a plot of the sensitivity against the false-positive rate for the evaluation of the KLR, is shown in Figure 5. From the ROC curve, we calculated the area under the curve (AUC), which is a threshold-independent measure. An AUC value above 0.7 is an indication of a useful prediction, and a good prediction method achieves a value above 0.85 [31]. NetTurnP, DEBT, E-SSpred, and SVM achieved AUC of 0.864, 0.84, 0.84, and 0.87, respectively; our method achieves AUC of 0.861.

## 4. Discussion

Prediction of *β*-turns has attracted researchers interest because it plays the following important roles.

*β*-turns have been proposed to be important in folding because they are capable of initiating productive structure formation without a large loss in chain entropy since the interactions involved in turn formation are largely local [32]. They can play two different roles in the folding reaction of a protein. They can be either folding-active elements and function as initiation sites or folding-passive elements that form only after other regions develop. These different roles are likely to arise from the relative importance of the various interactions in forming the native states of different proteins [33].

Turns can influence the stability of a protein's native state both by their intrinsic preference to sample favorable space and by their side-chain packing interactions and local environment [34]. Since *β*-turns usually occur on the exposed surface of a protein, they are well suited to participate in ligand binding, molecular recognition, protein-protein, or protein-nucleic acid interactions, thus modulating protein functions and intermolecular interactions. Additionally, they are frequent sites of posttranslational modifications such as phosphorylation and glycosylation, which are used to tune interactions [4].

*β*-turns are also involved in the biological activity of peptides as the bioactive structures that interact with other molecules such as receptors, enzymes, or antibodies. Recent years have seen interest in mimicking *β*-turns for the synthesis of medicines. Thus, *β*-turn is an important component of protein structure whose prediction can provide enormous information to the researchers working in the field of drug design. So the prediction of *β*-turns would not only aid in overall tertiary structure prediction but also assists in fold recognition studies.

Throughout the previous research on *β*-turns prediction, predictors based on machine-learning method emphasize selecting proper features to improve prediction performance. Secondary structures and PSSMs are widely used in the predictions and have been proven to be the most helpful features. Using these features the proposed KLR method achieves comparable results to the SVMs methods. To design a method that can be applied in *β*-turn prediction, there are four main concerns. These concerns are (1) the size of the dataset, (2) the need for dealing with input examples of variable length, (3) the need to have probabilistic outcomes, and (4) the need to perform multiclass classification. When the dataset is very large such as the *β*-turns data, people neglect the last two concerns and concentrate on selecting a classifier that deals with large datasets effectively. Since SVMs methods are designed in a way that can handle large-scale datasets, they become the choice for most of the *β*-turns classification methods. However, SVMs do not address the last two concerns directly. KLR is not used in large-scale datasets such as *β*-turns data classification although it provides elegant solution to the last two concerns, simply because it is inapplicable in such datasets. The last two concerns are very important for *β*-turns classification, since there is a need for multiclass classification for the *β*-turns type. FS-KLR extends the applicability of KLR for large-scale datasets. This way it can address all of the aforementioned concerns.

## 5. Conclusion

 In this paper, we presented sparse KLR method for *β*-turns prediction. Our method is based on FS-KLR in which trust region Newton's method for large-scale LR is used as a basis to solve the approximate problem, while Nystrom method is used to approximate the features' matrix. Our method uses secondary structure information and PSSMs as input features. Empirical evaluations using three nonredundant datasets show that our predictions provide favorable *Q*_total_, *Q*_observed_, and MCC when compared with the state-of-the-art methods that used secondary structure information and PSSMs as features. Using our method we achieved *Q*_total_ and MCC of 80.7% and 0.50, respectively, on BT426 dataset. In addition, KLR yields probabilistic outputs and its extension to the multiclass case is well defined, which will be appropriate for *β*-turns types prediction. The computational complexity of our method is *O*(nm^2^) and its computation time is by far less than that of SVMs methods.

## Figures and Tables

**Table 1:** *Q*_total_ and MCC for different values of selected vectors *m*.

Number of selected vectors *l*	*Q*_total_	MCC
70	79.96	0.46
80	80.32	0.47
90	80.25	0.47
100	80.38	0.47
110	80.41	0.47
120	80.54	0.48
130	80.51	0.48

**Table 2:** Comparison of KLR with other recent *β*-turns prediction methods on BT426 dataset.

Method	*Q*_total_	*Q*_pred_	*Q*_obs_	Specificity	MCC
KLR	80.7	58.98	65.25	85.34	0.50
BTNpred [6]	80.9	62.7	55.6	N/A	0.47
NetTurnP [13]	78.2	54.4	75.6	79.1	0.50
BetaTPred2 [10]	75.5	49.8	72.3	N/A	0.43
BTPRED [9]	74.9	55.3	48.0	N/A	0.35
DEBT [8]	79.2	54.8	70.1	N/A	0.48
SVM [26]	79.8	55.6	68.9	N/A	0.47
BTSVM [27]	78.7	56.0	62.0	N/A	0.45
E-SSpred [28]	80.9	63.6	49.2	N/A	0.44
1–4 & 2-3 correlation model [29]	59.1	32.4	61.9	N/A	0.17

**Table 3:** Comparison of KLR with other recent *β*-turns prediction methods on BT547 and BT823 datasets.

Method	Dataset	*Q*_total_	*Q*_pred_	*Q*_obs_	MCC
KLR	BT547	80.46	59.04	65.36	0.50
BTNpred		80.5	61.6	54.2	0.45
COUDES [18]		74.6	48.7	70.4	0.42
SVM [26]		76.6	47.6	70.2	0.43

KLR	BT823	80.66	58.42	64.64	0.49
BTNpred		80.6	60.8	54.6	0.45
COUDES		74.2	47.5	69.6	0.41
SVM [26]		76.8	53.0	72.3	0.45

**Table 4:** Comparison of the elapsed time in seconds between KLR, BTNpred, and E-SSpred.

Dataset	KLR	BTNpred	E-SSpred
BT426	753.55	11077.185	13036.415
BT547	940.55	13261.755	15726.2
BT823	683.44	18183.256	24140.072
